# Proceedings of Ninth Annual Session International Hahnemannian Association

**Published:** 1888-08

**Authors:** 


### PROCEEDINGS OF THE NINTH ANNUAL SESSION OF THE INTERNATIONAL HAHNEMANNIAN ASSOCIATION.

The ninth annual session of the International Hahnemannian Association was called to order at the International Hotel, Niagara Falls, at eleven A. M., June the 19th, by the President, Dr. William P. Wesselhœft. The President then delivered his address, which, being an exceedingly able and interesting paper, was listened to with the closest attention. (This address is given in full in this issue.)

The Secretary, Dr. Ballard, then read his report of the work done during the past year; report was referred to a committee.

The Treasurer, Dr. Hawley, then presented his report, which was received and referred to an auditing committee.

Letters of regret at their absence from the meeting were read from Drs. O. P. Baer, G. W. Sherbino, and G. H. Clark.

The report of the Board of Censors was then presented by their efficient Chairman, Dr. J. A. Biegler. A motion was made and carried that the Secretary should cast an affirmative vote for such applicants for membership as were recommended by the Board. The following were elected to membership: C. Baldilli, M. D., of Florence, Italy; Caroline E. Hastings, M. D., of Boston; William A. D. Pierce, M. D., of Philadelphia; Clarence C. Howard, M. D., of New York; Robert N. Fallon, M. D., of Clifton, England; Stuart Close, M. D., of Brooklyn; R. B. Johnstone, M. D., of Philadelphia; F. L. McIntosh, M. D., of Melrose, Mass.

The first regular Bureau to report was the Bureau of Homœopathic Philosophy, Dr. J. T. Kent, Chairman. This is the new Bureau added to their work by the Association last year. Upon the suggestion of Dr. Kent, as was most fit, Dr. Ad.

Lippe was made the first Chairman of this new Bureau; after the death of Dr. Lippe, Dr. Kent was appointed Chairman.

In his arrangement of the work for the "Bureau of Homœopathic Philosophy" for this year, it had been the purpose of the late Dr. Lippe to have had read a series of three papers on these very important points in homœopathic practice:

The first, on "How to Examine the Patient," by Dr. P. P. Wells; the second, on "How to Select the Homœopathic Remedy," by Dr. Lippe himself; the third, on "The Second Prescription," by Dr. J. T. Kent.

In this way it had been the desire of our lately deceased friend, the former Chairman of this Bureau, to cover by a connected series of papers three cardinal features of homœopathic practice; three points which, if well understood by every prescriber, would render practice easier and success more certain. By the unfortunate death of our great colleague we are deprived of much; not the least of our loss we may consider the failure to secure from his ripe experience words of counsel on the important question of "How to Select the Homœopathic Remedy." Under this Bureau the first paper read was that of our veteran friend, Dr. P. P. Wells, upon "The Examination of the Patient." It is needless to add that the paper was a most thorough exposition of the objects and aim of the practitioner in performing this most important of all his duties. The Doctor showed how this work was to be done and what use was to be made of the work when it was done. This paper was listened to with the greatest interest and attention throughout. When the Doctor had finished its reading a spirited discussion ensued, which was participated in by Drs. Allen, Ballard, Gee, Hawley, Kent, Biegler, Butler, Nash, and Wesselhœft.

Dr. Edward Cranch presented a paper upon "Characteristic Symptoms, Section 153," which is given in full elsewhere in this issue. Dr. W. S. Gee gave a dissertation upon "Section 3," which is, perhaps, one of the most difficult paragraphs in the *Organon* to discuss. The paper was commented upon by Drs. Allen, Biegler, Holmes, Butler, Nash, and Wesselhœft. Dr. Kent then read his paper upon "The Second Prescription," which was discussed by Drs. Holmes, Hitchcock, Hawley, J. V. Allen, Biegler, Wesselhœft, Ballard, Campbell, Nash, and Butler. Dr. Wesselhœft considered it a very valuable paper, and one which confirmed many of his own personal experiences. (We also publish this paper in full in this issue.)

Continuing the work of the Bureau of Homœopathic Philosophy, Dr. Kent stated that he had a voluntary contribution from a physician who was not a member of the Association, but that he considered the paper so well worth their hearing that he hoped some one would offer a resolution that the paper be read. Such a motion was made and seconded. The paper in question was one upon "Sycosis," by Dr. S. L. G. Leggett, which the Doctor was requested to read.

Dr. Wesselhœft commended the paper and hoped it would be fully discussed, which was done by Drs. Gee, Stowe, Allen, Butler, Kent, and others.

The next Bureau to report was that of "Materia Medica and Provings." This is generally the most important work of the Association, and as this year the Bureau was presided over by our able friend, Dr. Gee, and contained such laborers as Drs. Wells, Kent, Butler, Allen, Holmes, Sawyer, Hitchcock, Lawton, Nash, Guernsey, Clark, its reputation for good work was certainly well-sustained. The first paper read was one by Dr. Kent, entitled "A Study of our Materia Medica," illustrated by a partial proving of Lachesis. The paper was exhaustively discussed by Drs. Hawley, Wells, Stowe, Biegler, Butler, Hitchcock, Custis, and Nash.

Dr. H. C. Allen next read a very useful paper giving the results of a proving of Magnesia phosphoricum. The President expressed great pleasure in Dr. Allen's paper and proving; said he believed Magn.-phos. would prove to be one of the great remedies for the removal of woman's sufferings, not only during menstruation but at a great many other periods.

Dr. Allen suggested that the women of the Association should assist in developing this remedy, whereupon several of the lady members volunteered to assist Dr. Allen in his provings. It is to be hoped this good work will go on, for the weak spot in our provings is the lack of female provers.

Upon the motion of Dr. Emory the election of officers for the ensuing year was made the first business for the afternoon session of June 21st. Dr. H. P. Holmes then read a fine paper upon convulsions, which subject was further developed in discussion by Drs. Emory, Custis, Wesselhœft, Ballard, and Hitchcock. Dr. C. H. Lawton then presented a paper; afterward Dr. E. Rushmore gave one of his good papers upon Belladonna and Rhus toxicodendron, which was also discussed by the members.

On the opening of the morning session of June 21st, Dr. E. B. Nash read a paper narrating some clinical experiences, which was discussed by Drs. Allen, Kent, and Wells. Dr. J. A. Biegler then gave an essay upon "Mental Alienation," showing what true Homœopathy could do in such cases. Its interest was added to further by Drs. Stowe, Wells, and Kent in the discussion which followed.

Dr. Biegler also read a paper upon "Kali-phos. in Acute Laryngitis," which was discussed by Drs. Gee, Allen, and Wells.

Dr. Clarence Willard Butler next read one of his interesting papers upon "Experiences with Synapis Nigra." Dr. William A. Hawley then presented a paper narrating a "Case of Cancer of the Stomach Cured by Arsenicum," which was discussed by Drs. Gee, Hitchcock, Biegler, Holmes, and Wells.

At the afternoon session, as per motion of previous day, the election of officers was taken up. Great modesty was suddenly developed; each member desired some one else to gain the honors. The final outcome of the election was the selection of the veteran William A. Hawley, M. D., for President; Dr. W. S. Gee, for Vice-President; our energetic young friend, S. A. Kimball, M. D., was chosen Secretary, and he will make a good one; Dr. Tyrrell, of Toronto, was elected Treasurer. Upon motion of Dr. Ballard, the old reliable Board of Censors were continued for a third term; the Board is composed of Drs. Biegler, Bell, Gee, Rushmore, and Butler.

The selection of next place of meeting gave rise to considerable discussion, for, as usual, nearly every member had a favorite place. Toronto was finally chosen, which will be the first session of the Association outside of this country, unless the meeting at Long Branch, N. J., be so considered.

Under the active work of Dr. Alice B. Campbell the Bureau of Clinical Medicine made a good report. Dr. H. P. Holmes read a paper, which was commented upon by Dr. Wells. Dr. E. W. Sawyer gave a paper upon "A Few Cases of Cancer," which Drs. Carleton and Wells discussed. The President, Dr. Wesselhœft, then read a paper upon "Aloes," which was practical and interesting; it was discussed by Drs. Allen, Cash, Sawyer, Nash, Butler, Ballard, Custis, Stowe, Wells, and Carleton. Dr. Campbell then read the notes of a case from her own experience, which Drs. Holmes, Gee, Wells, and Allen discussed. Dr. J. V. Allen next read a paper upon "Carcinoma," which excited an interesting discussion. Dr. Pease presented a paper upon the "Hypertrophy of the Prostate." Dr. George H. Clark, of Philadelphia, read a paper upon "Some Symptoms of Sycosis." The paper will be found in full in this number.

June 22d the Bureau of Obstetrics, through Dr. E. P. Hussey, its Chairman, presented its report. Dr. J. B. G. Custis read a paper; he was followed by Dr. Frank Powel on "A Peculiar

Case," which was discussed by Drs. Gee, Allen, Campbell, Wells, and Holmes. Dr. Flora A. Waddell next presented a paper on the "Diseases of Women."

The Bureau of Surgery, etc., was represented by Dr. J. B. Bell, Chairman, with Drs. Edmund Carleton, Thomas L. Dillingham, Julius Schmitt, G. G. Gale, and E. A. Ballard. This is an important Bureau in the I. H. A., as it endeavors to show how the law of similars aids the surgeon; how it removes in many cases the necessity for the use of the knife, and also how, in cases where operations must be performed, it saves the patient from dangerous sequelæ. It is an important work, and should not be slighted. Under homœopathic medication many surgical operations are rendered needless, and many operations are made possible where, under allopathic treatment, death would surely follow.

After the usual routine business the Association adjourned *sine die*. The session seems to have been one of the most useful and active the I. H. A. has ever held, and encourages all to work for the advancement of their beloved science of Homœopathy.

---

